# Magnetic Resonance Study of Bulky CVD Diamond Disc

**DOI:** 10.3390/ma17081871

**Published:** 2024-04-18

**Authors:** Alexander Shames, Alexander Panich, Lonia Friedlander, Haim Cohen, James Butler, Raymond Moreh

**Affiliations:** 1 Department of Physics, Ben-Gurion University of the Negev, Beer-Sheva 8410501, Israel; sham@bgu.ac.il (A.S.);; 2 Ilse Katz Institute for Nano-Scale Science and Technology, Ben-Gurion University of the Negev, Beer-Sheva 8410501, Israel; 3 Department of Chemical Sciences, Faculty of Exact Science, Ariel University, Ariel 4077625, Israel; 4 Cubic Carbon Ceramics, Huntingtown, MD 20639, USA

**Keywords:** CVD diamond, electron paramagnetic resonance, nuclear magnetic resonance, paramagnetic defects, defects distribution, spin–lattice relaxation time

## Abstract

Diamonds produced using chemical vapor deposition (CVD) have found many applications in various fields of science and technology. Many applications involve polycrystalline CVD diamond films of micron thicknesses. However, a variety of optical, thermal, mechanical, and radiation sensing applications require more bulky CVD diamond samples. We report the results of a magnetic resonance and structural study of a thick, sizable polycrystalline CVD diamond disc, both as-prepared and treated with e-beam irradiation/high-temperature annealing, as well as gamma irradiation. The combination of various magnetic resonance techniques reveals and enables the attribution of a plentiful collection of paramagnetic defects of doublet and triplet spin origin. Analysis of spectra, electron, and nuclear spin relaxation, as well as nuclear spin diffusion, supports the conclusion of significant macro- and micro-inhomogeneities in the distribution of nitrogen-related defects.

## 1. Introduction

Chemical vapor deposition (CVD) diamond is a material that has found applications in numerous fields. They include optical applications (infrared windows, lenses, ATR units, X-ray windows), thermal applications (heat spreaders, laser sub mounts, X-ray targets), mechanical applications (cutting tools, scalpels, knives, length gauge tips, wear-resistant components, e.g., for textile machines, inserts for dresser tools), electrochemical applications (electrodes, electrochemical detectors, biochemical sensors), and radiation sensors (ionizing radiation detectors/dosimeters, fluorescence beam monitors). While natural and high pressure–high temperature (HPHT) synthetic diamonds are grown at high pressures under conditions in which diamond is the thermodynamically stable form of crystalline carbon, the growth of CVD diamond occurs at a low pressure in which diamond is not thermodynamically stable, and its growth occurs under highly nonequilibrium conditions [1,2]. Conditions and chemistry of the growth media for CVD diamond are also different from that in which HPHT synthetic diamonds and natural diamonds grow, as CVD diamond is grown at low temperatures and pressures from a mixture of hydrogen and a carbon-containing gas (typically CH_4_ or other hydrocarbons) activated by a hot filament, microwave plasma, or other means.

Many important properties of diamonds depend on paramagnetic lattice defects. These defects can result from three basic components, as well as their combinations, including (i) substitutional impurity as the presence of a different atom at a host lattice site, (ii) vacancy as the absence of a host atom at a lattice site, and (iii) interstitial as the presence of a host or impurity atom not at a lattice site. The defects occur during preparation or may be intentionally (or accidentally) introduced by displacing carbon atoms through ionizing irradiation [3,4] or doping. Even at low concentrations (parts per million, ppm), point defects may significantly affect the properties of diamonds. The abovementioned paramagnetic defects make diamonds show great potential for a variety of applications, particularly, NV^−^ centers may work as basic units of a quantum computer and have potential applications in novel electronics and computational science, including quantum cryptography and spintronics [5]. Therefore, the study of these defects is of great importance. The defects can be detected using different types of spectroscopy, including electron paramagnetic resonance (EPR), photoluminescence, cathodoluminescence, and light absorption in the infrared, visible, and ultraviolet parts of the spectrum. Among these, the EPR technique is the most informative.

The first EPR study of CVD diamonds was reported in 1988 [6]. Since that work, both EPR and electron nuclear double resonance (ENDOR) have been extensively used for revealing structure and defects in a variety of CVD diamond syntheses [7,8,9,10,11,12]. Several paramagnetic defects have been discovered, of which the most common are defects formed by nitrogen impurities, as well as by hydrogen atoms located close to carbon dangling bonds in grain boundaries. The appearance of many defects depends on the preparation procedure and post-processing of samples of CVD diamond. As CVD diamonds are increasingly used in many areas of research and industry, detailed information on the appearance of the defects and their transformations in CVD diamonds remains an important issue.

In many samples grown for both research and applications, relatively thin (up to tens of microns) CVD diamond films are considered [13,14,15,16,17,18,19,20,21,22,23,24]. In the future, a variety of optical, thermal, mechanical, and radiation sensor applications will require more substantial CVD diamonds [25,26,27,28,29,30,31,32]. It was of certain interest to probe the quality and defect structure of such material. To investigate the properties of such a material at the atomic level, we present in this article the results of EPR, nuclear magnetic resonance (NMR), Fourier transform infrared (FTIR) spectroscopy, and X-ray diffraction (XRD) investigations of a thick, sizable CVD diamond disc. The measurements were carried out using both the as-prepared disc and after e-beam and gamma irradiation, followed by annealing. Detailed investigations of paramagnetic defects have been carried out using a variety of continuous wave (CW) EPR techniques. Several types of primary doublet (*S* = 1/2) and triplet (*S* = 1) defects are revealed, attributed, and quantified. Both EPR and NMR measurements show an inhomogeneous distribution of nitrogen-related defects across the disc at both macro- and micro-levels. NMR data correlate with the EPR results and reveal the nuclear spin diffusion throughout the sample. XRD provides bulk structural information and indicates that the CVD diamond disk contains grains with a distinct preferred growth direction (111).

## 2. Materials and Methods

CVD diamond was deposited from gas-phase mixtures containing hydrogen and methane. The sample was grown in a home-built microwave plasma system made of ASTeX components with a 5 kW microwave source at 2.45 GHz using an HPMM (cylindrical style) chamber, with a water-cooled substrate holder, on a molybdenum substrate (in 1996 at the Naval Research Laboratory). It was approximately 2” in diameter. The final sample was a disc with a diameter of 50 mm and thickness of ca. 560 microns; the plate was delaminated from the substrate on cool down. There were 2 stages to growth, nucleation and then growth. The preparation conditions are recorded in Table 1. Defect/impurity incorporation during growth depends on the local stereochemistry of the growth surfaces, generally, step roughened (100), (111), and (113); (110) apparent surfaces are generally micro-faceted with (111) planes.

Room temperature (RT, *T* ≈ 295 K) continuous wave (CW) EPR experiments were carried out using a Bruker (Billerica, MA, USA) EMX220 X-band (ν~9.4 GHz) EPR spectrometer equipped with an Oxford Instrument (Abingdon, UK) ESR900 cryostat and an Agilent (Santa Clara, CA, USA) 53150A frequency counter. Single (10–15 mg weight) and multiple (50–120 mg of total weight) pieces of defragmented large CVD diamond disc were placed in 4 mm o.d. Wilmad EPR quartz tubes and fixed into the Bruker ER 4102ST cavity. Accurate determination of the electronic *g*-factors and densities *N*_s_ of primary paramagnetic species within the *g* = 2.00 region was accomplished by a comparison to a reference sample of highly purified detonation nanodiamond (DND) powder with *g* = 2.0028 ± 0.0002 and *N*_s_ = 6.3 × 10^19^ spins/g or 1255 ppm [33]. The quantification of primary species was carried out as described earlier [34]. Estimation of the triplet centers content and accurate determination of the effective *g*-factor for the half-field lines were performed using a comparison of the double-integrated intensities of the half-field (*g*~4.00 region) EPR lines of all samples under investigation to those for the fluorescent microdiamond sample FMD having NV^−^ (W15) content of 5.4 × 10^17^ spin/g or 10.7 ppm and *g*_eff_ = 4.272 (by EasySpin simulation for ν = 9.463 GHz) [35,36]. Typical experimental error in the determination of spin densities does not exceed 15%.

EPR spectra were registered in two modes, including (a) conventional slow passage (SP) and (b) fast passage (FP) spectra. SP spectra were detected in-phase as the first derivative of the absorption lines using sweep rate SR = 0.57 mT/s and 100 kHz magnetic field modulation amplitude A_m_ = 20 μT for the spectra of primary centers, as well as SR = 7.6 mT/s and A_m_ = 75 μT for the triplet centers. FP spectra of primary centers were detected out-of-phase (90°, 270°) as absorption lines using SR = 9.2 mT/s and A_m_ = 5 μT. Microwave power saturation experiments for primary centers were performed by sweeping the incident microwave power level *P*_MW_ incremented from *P*_MW_ = 200 nW (60 dB) to *P*_MW_ = 200 mW (0 dB) in 2 dB steps. In situ illumination of the samples was performed through the irradiation grid of the Bruker ER 4102ST cavity with a Fiber-Lite Model 3100 light source (CW, 30 W). EPR data processing and spectral simulation were carried out using Bruker WIN-EPR/SimFonia, EasySpin [36], and OriginLab 7.0 software packages.

For the NMR measurements, similar multiple pieces of defragmented large CVD diamond disk, taken from its two different parts, were used. RT ^13^C NMR measurements were carried out using a pulse solid-state NMR spectrometer (Tecmag, Inc., Houston, TX, USA), equipped with a homebuilt NMR probe and an Oxford Instruments wide bore superconducting magnet. The measurements were carried out in a static mode in the external magnetic field *B*_0_ = 8.0 T, corresponding to the ^13^C resonance frequency of 85.62 MHz. The ^13^C spectra were measured using the Hahn echo pulse sequence ${\left(\frac{\pi }{2}\right)}_{x}-\tau_{d}-{\left(\pi \right)}_{x}$, and the ^13^C spin–lattice relaxation times *T*_1n_ were measured using a saturating comb pulse sequence ($\frac{\pi }{2}$ pulse train). Magnetization recovery in measuring *T*_1n_ was fitted by an exponential function. The duration of the $\frac{\pi }{2}$ pulse was 4.6 μs. Data processing and simulations were performed using the OriginLab 7.0 software.

In addition, static RT ^1^H NMR measurements were carried out using the same spectrometer and magnet at the ^1^H resonance frequency of 340.5 MHz. The spectra were measured using the solid echo pulse sequence ${\left(\frac{\pi }{2}\right)}_{0^{{}^{\circ}}}-\tau_{d}-{\left(\frac{\pi }{2}\right)}_{90^{{}^{\circ}}}$, a technique that refocuses homonuclear dipole–dipole couplings between protons (here subscripts denote the phases of pulses). The duration of the π/2 pulse was 2.2 μs.

FTIR spectra analysis in the range of 500–4000 cm^−1^ were carried out with a Bruker Alpha2 FTIR spectrophotometer, equipped with a ZnSe beam splitter, with a resolution of 2 cm^−1^, and collecting 24 scans for each sample. The diamond was placed in a transmittance plug in unit to the FTIR unit. The concentration of the different nitrogen species in the diamonds was calculated using FTIR spectroscopy upon evaluating the C–N bond absorption in the 1000–1400 cm^−1^ region of the infrared spectrum [37]. Nitrogen concentration was estimated using QUIDDIT program published by Spiech [38].

XRD measurements were conducted at the Ilse Katz Institute for Nano-scale Science and Technology at Ben-Gurion University of the Negev. The measurements were collected in Bragg–Brentano symmetric (θ/2θ) geometry using a Panalytical Empyrean III (Malvern Panalytical B.V., Brussels, Belgium) multipurpose diffractometer equipped with a Cu X-ray source (K_a_ radiation, λ = 1.541 Ǻ), operated at v = 45 kV, I = 40 mA. The instrument was further equipped with the iCore–dCore automated XRD optics combination, a pixCEL 3D detector in 1D line-detector mode, and an automated 3-axis eulerian cradle sample stage with control along the *z*-axis (height) and sample rotation (Φ) and tilt (χ) angles. The CVD diamond sample was measured twice. The first measurement was collected with no sample tilt (χ = 0°). The second measurement was collected at a tilt optimized to maximize the intensity of the (220) reflection (χ = 45.76°). Collected data were analyzed for phase identification and crystallographic direction by comparison to ideal diamond diffraction patterns found in the International Center for Diffraction Data (ICDD) Powder Diffraction File (PDF-4+) database (2022). The full-width half maximum (FWHM) of relevant reflections were calculated using the Full-Prof Suite Toolbar (version July 2017) to enable the use of the Scherrer formula to estimate crystallite size.

All measurements were performed with as-received samples and those e-beam irradiated with the dose of 1 kGy and gamma-irradiated with the dose of 2 MGy. Irradiated samples were also annealed for 2 h in a vacuum at a temperature of 850 °C.

## 3. Results

### 3.1. XRD Data

**Figure 1:**
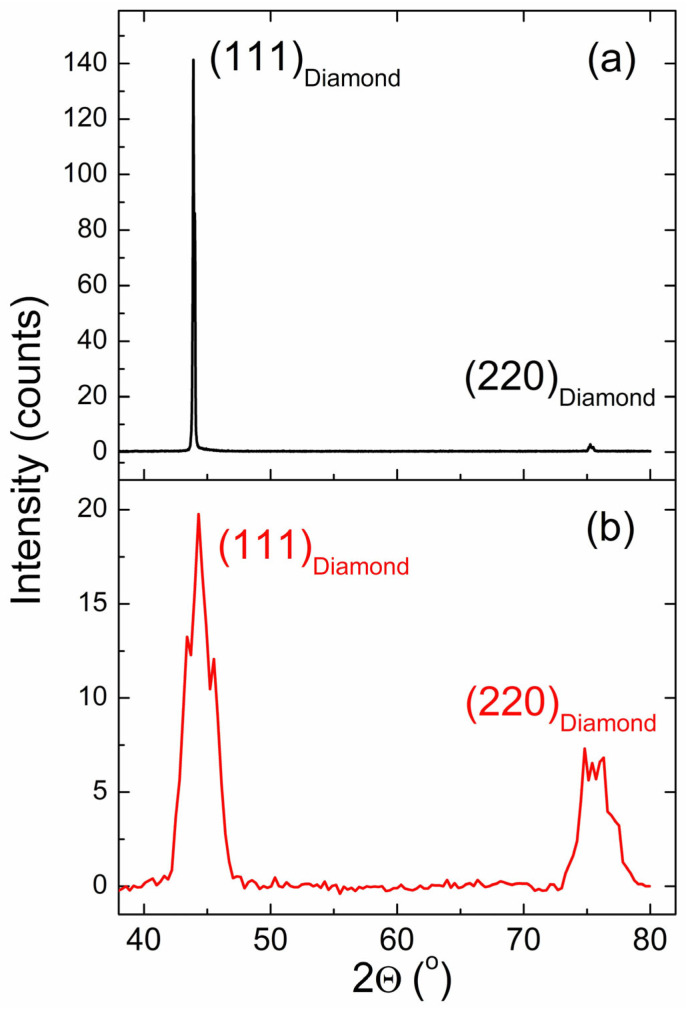
Bragg–Brentano geometry reflectance XRD measurements at no tilt (**a**) and with tilt optimized to maximize the intensity of the (220) reflection (**b**). The optimized sample tilt in (**b**) is χ = 45.76°.

The XRD pattern of the investigated CVD diamond (Figure 1) displayed diffraction maxima at the angles 2θ = 43.9° and 75.2°, corresponding to the (111) and (220) reflections of a cubic (Fd3m) diamond lattice, respectively (Figure 1). The presence of diffraction peaks from two crystallographic directions suggests a polycrystalline sample because multiple crystallites must be present to meet the Bragg condition in two different orientations from within the sample, which was a solid disc. The initial measurement of the flat (χ = 0°) CVD diamond disk shows a relative intensity ratio between the (111) and the (220) reflections that is more than 20 times greater than expected (ideal) for a cubic diamond lattice. The (111) peak intensity suggests greater than micrometer-sized crystallites (calculated Scherrer <d> > 1220 nm) in this growth direction and a strong preferred orientation for the (111) crystallographic plane (Figure 1a). In contrast, the FWHM of the un-tilted (χ = 0°) (220) reflection suggests a crystallite <d>~90 nm. Optimizing and repeating the XRD measurement oriented around the (220) reflection, returns the relative intensity ratio between the (111) and (220) reflections to the expected range; the (220) reflection is roughly 30% of the (111) intensity (Figure 1b). Maximizing the (220) intensity to optimize sample tilt and eliminate the effects of preferred orientation produces reflections with FWHM values that indicate an overall crystallite size of ~4 nm. This suggests that crystallite diameters in the (220) direction range from 4 to 90 nm, significantly smaller than those observed for the preferred (111) direction.

### 3.2. FTIR Spectra

**Figure 2:**
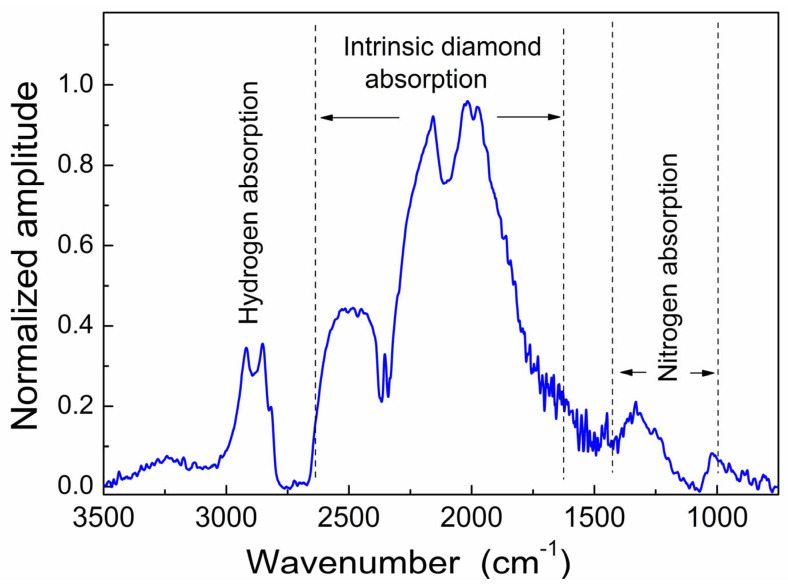
Experimental FTIR spectrum of the CVD diamond.

FTIR spectra (Figure 2) are dominated by phonon absorption bands in the range of 1500–2600 cm^−1^, which are intrinsic to pure diamond. The peaks at 2800–3000 cm^−1^ correspond to the stretching vibration of C–H bonds; this finding correlates with the below (Section 3.4) NMR data revealing the proton resonance signal of C–H bonds. A region between 1500 and 1000 cm^−1^ includes the absorption from impurity activated single phonons (nitrogen). The spectra do not show visible changes under irradiation and annealing.

The FTIR spectrum indicates the presence of two types of nitrogen atom impurities in the CVD diamond, including A-centers (N2 atoms) and C-centers (single nitrogen atoms). Broad absorption lines do not allow reliable quantitative estimation of each type of nitrogen defects. The total amount of nitrogen in this CVD sample does not exceed 90 ppm.

### 3.3. EPR Data

General view RT EPR spectra of all the samples under study consist of a low-intensity broad signal attributed to trace amounts of metallic impurities (further characterization of such impurities falls outside the scope of this study), intense sharp lines within the *g* = 2.00 region arising from the paramagnetic defects with *S* = 1/2, and weak narrow EPR signals observed within in the *g*_eff_ = 1.8, 2.4, and 4.00 regions associated with “allowed” ∆*M*_S_ = 1 and “forbidden” ∆*M*_S_ = 2 lines in EPR spectra of triplet (*S* = 1) centers. Because no changes in EPR spectra were revealed in the pieces of the initial CVD sample which had undergone e-beam and gamma irradiation and further high-temperature annealing, the results presented here will describe the untreated (pristine) CVD diamond. EPR spectra of both single and multiple pieces demonstrate the same orientational-independent polycrystalline-like patterns. Because samples consisting of multiple randomly oriented pieces provide higher signal-to-noise ratios (which are extremely important for the reliable registration of weak triplet-related signals) the results presented were collected just on these samples of total weights 50–120 mg.

#### 3.3.1. EPR Spectra of Primary Paramagnetic Centers

**Figure 3:**
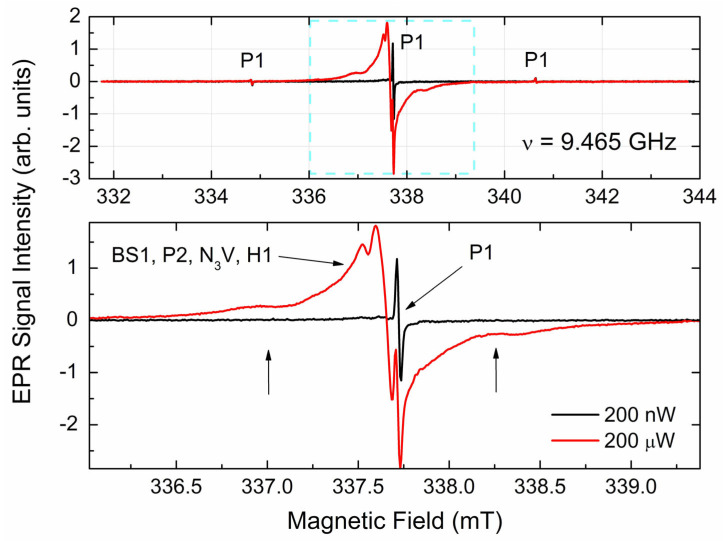
RT SP EPR spectra of primary species in the pristine CVD diamond samples. Spectra recorded at *P*_MW_ = 200 nW (black trace) and *P*_MW_ = 200 μW (red trace), SR = 0.57 mT/s, A_m_ = 20 μT, receiver gain RG = 2 × 10^5^, and number of coherent acquisitions n_acq_ = 100, ν = 9.465 GHz. Upper panel shows the entire pattern within the *g* = 2.00 region; sharp triplet indicates P1 centers; lower panel shows zoom of the central line; and arrows point out signatures attributed to various S = 1/2 species (see the text). Vertical arrows point out “forbidden” hyperfine lines in EPR spectra of H1 centers, observed at high *P*_MW_ levels.

Figure 3 shows the *g* = 2.00 region EPR spectra recorded at two levels of the incident power.

**Figure 4:**
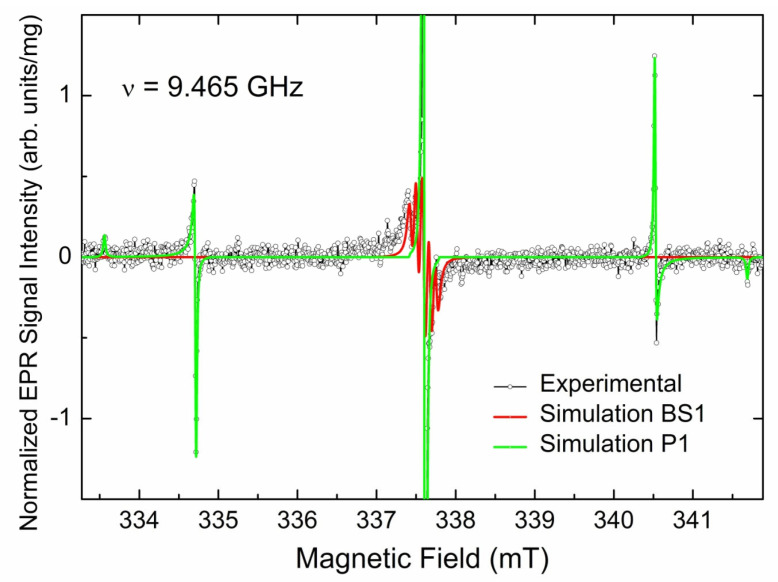
Vertical zoom of the RT SP EPR spectrum in Figure 3 recorded at *P*_MW_ = 200 nW; black open circles—experimental spectrum, red trace—simulated spectrum of BS1, green trace—simulated spectrum of P1. Spin–Hamiltonian parameters used for simulation are listed in the text.

SP EPR spectrum observed at low incident power (Figure 3 black trace) clearly demonstrates sharp orientation independent hyperfine triplet pattern, which is characteristic of P1 centers [39,40] overlapped with some broad signals for both central component and satellites. Sharp P1 components start being saturated at 5 μW, whereas other components become saturated at higher power levels. Thus, the spectrum observed at 200 μW (Figure 3, red trace) reveals broad lines around the central (*m*_I_ = 0) P1 line, as well as broad components around satellite (*m*_I_ = ±1) P1 lines. The non-saturated spectrum (Figure 4, black open circles) may be reasonably simulated as superposition of two *S* = 1/2 paramagnetic centers, as follows: P1 (single ^14^N with *I* = 1, *g*_iso_ = 2.0024, *A*_||_ = 4.064 mT, *A*_⊥_ = 2.905 mT, peak-to-peak Lorentzian line width ∆*H*_pp_(Lorentz) = 0.018 mT—see Figure 4, green trace) and an a center designated as BS1 (it is generally accepted to designate new paramagnetic defects in diamonds by abbreviations of the laboratory’s location; in this case, BS means Beer-Sheva), characterized by the electronic spin interacting with two *I* = 1 nuclei (*g*_iso_ = 2.0028, *A*_iso_ = 0.08 mT, ∆*H*_pp_(Lorentz) = 0.05 mT—see Figure 3, red trace).

The total content of primary paramagnetic centers, obtained from non-saturated SP EPR spectra was found to be sample-dependent (because different samples originated from different parts of the initial CVD diamond disk) and varies from 2.3 ppm to 4.9 ppm; among these, P1 only content varies from 0.6 ppm to 1.1 ppm.

**Figure 5:**
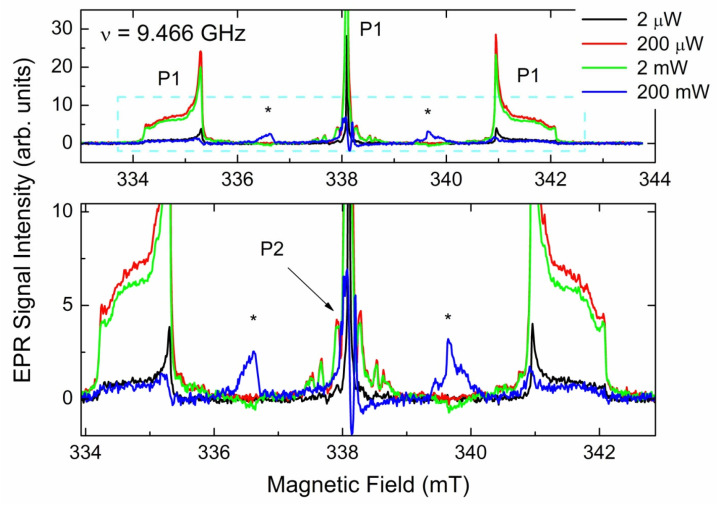
RT FP EPR spectra of primary species in the pristine CVD diamond samples. Spectra recorded at SR = 9.2 mT/s, A_m_ = 5 μT, RG = 1 × 10^5^, n_acq_ = 100, ν = 9.466 GHz. Spectra were recorded at *P*_MW_ = 2 μW (black trace), 200 μW (red trace), 2 mW (green trace), and 200 mW (blue trace). Lower panel shows vertical zoom of satellite lines. Central P1 line is cut for the better presentation of satellite signals. Asterisks point out additional hyperfine satellite lines in between conventional P1 hyperfine components.

Further elucidation of the origin of other primary centers was performed using differences in saturation behavior of different primary paramagnetic defects. In fact, defects having longer electronic spin–lattice (T_eSL_) and spin–spin (T_eSS_) relaxation times (like those narrow P1 lines simulated in Figure 4) become saturated at lower microwave power levels, whereas BS1, P2 [39,40], and H1 [6,7,10] centers reveal quite strong signals at much higher power levels—see red traces in Figure 3. Moreover, it is well-known [39] that FP EPR spectra are extremely sensitive to paramagnetic centers having relatively long T_eSL_, at which the energy of the electron spin system is exchanged with the environment, and in homogeneously broadened lines [41]. Figure 5 represents the FP EPR spectra of one of the samples under study recorded at incrementing incident power levels.

Power-dependent FP EPR spectra clearly demonstrate that, in addition to easily saturating narrow P1 components, there are other P1 components that saturate at higher power levels. Some complicated, badly resolved hyperfine patterns on both sides of the central P1 line may be attributed to P2 centers [39,40], which were not observed in SP EPR spectra. Here, it is worth mentioning that FP EPR spectra show no lines that could be attributed to BS1 and H1 centers, which implies that these centers have significantly reduced relaxation times and makes them unobservable in FP detection mode. On the other hand, FP EPR spectra recorded at high power levels (*P*_MW_ > 10 mW) reveal two additional lines split by ~3 mT (asterisks in Figure 5) between the lines of the P1 hyperfine triplet. The origin of these lines will be discussed further.

#### 3.3.2. CW EPR Progressive Power Saturation of Primary Centers

**Figure 6:**
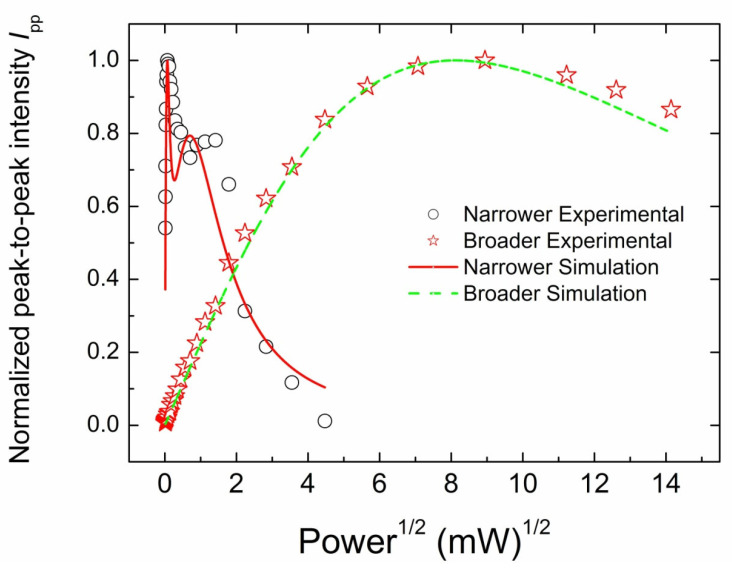
Peak-to-peak amplitudes of the central (*g* = 2.00) lines in the X-band SP EPR spectra of CVD diamond sample as a function of the incident microwave power (saturation curves); black open circles—narrow line, and red open stars—broad line. Line traces demonstrate best least square fits; red solid line—double component fit for the narrow line, green dashed line—single component fit for the broad line. At *P*_MW_ ≥ 20 mW the narrow line becomes totally unobservable.

Further evidence for the existence of several types of different paramagnetic centers contributing to the central line of CP EPR spectra in Figure 3 was obtained in progressive power saturation experiments. Figure 6 shows the dependence of the peak-to-peak amplitude of the central lines on the incident microwave power (plotted as *P*^1/2^)—saturation curves. The saturation of the narrow line reveals two maxima, including the sharp one characterized by the saturation onset 5 μW and the broad one at the onset of ~2 mW. In contrast, the saturation curve for the broad line demonstrates saturation onset at ~50 mW. Thus, the narrow line consists of two components, including very fast and moderate saturating ones. Both these components become totally saturated at *P*_MW_ ≥ 20 mW and, therefore, unobservable. On the other hand, the broad line reveals a single component saturating at a noticeably higher power level. Analysis of the saturation curves’ behavior [42,43] allows for the estimation of T_eSL_ values for each of the components described above—see solid and dashed lines in Figure 6 representing best least square fits. Thus, paramagnetic centers responsible for the narrow line may be characterized by T_eSL1_~230 μs and T_eSL2_~1.6 μs, whereas the broad line reveals T_eSL_~0.21 μs.

#### 3.3.3. EPR Spectra of Triplet (*S* = 1) Paramagnetic Centers

**Figure 7:**
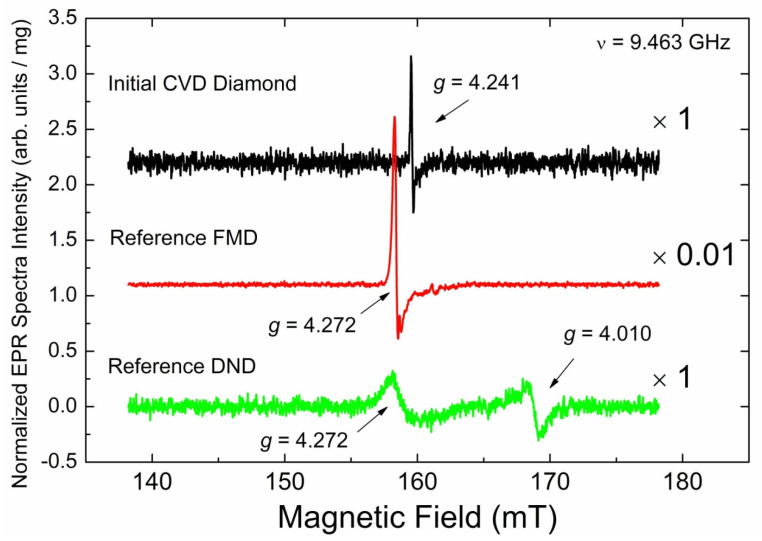
Half-field RT SP EPR spectra (*g* = 4.00 region); black trace—initial CVD diamond pieces, red trace—reference FMD sample, green trace—reference DND sample. Arrows mark effective *g*-factors for the “forbidden” lines with *g*_eff_ = 4.241 ± 0.005 (CVD), *g*_eff_ = 4.272 (FMD, DND) and *g*_eff_ = 4.010 (DND). Spectra recorded at SR = 7.6 mT/s, non-saturating power levels *P*_MW_ = 5 mW (CVD, DND), and *P*_MW_ = 50 μW (FMD), A_m_ = 0.075 mT (CVD, FMD) and A_m_ = 0.75 mT (DND), RG = 2 × 10^6^, n_acq_ = 400, ν = 9.463 GHz. Intensities of the spectra were normalized per unit mass and the uniform setup; scaling factors are shown on the right. Background signals have been subtracted.

In addition to the intensive signals within the *g* = 2.00 region, SP EPR spectra reveal a set of weak sharp signals within the half-field region *g*_eff_ = 4.00, as well as low- and high-filed satellites to the primary signals located at *g*_eff_ = 2.4 and 1.8, correspondingly. Figure 7 shows the half-field signal of the CVD diamond sample (black trace) in comparison with the half-filed signals in the reference FMD (red trace) and DND (green trace) samples.

**Figure 8:**
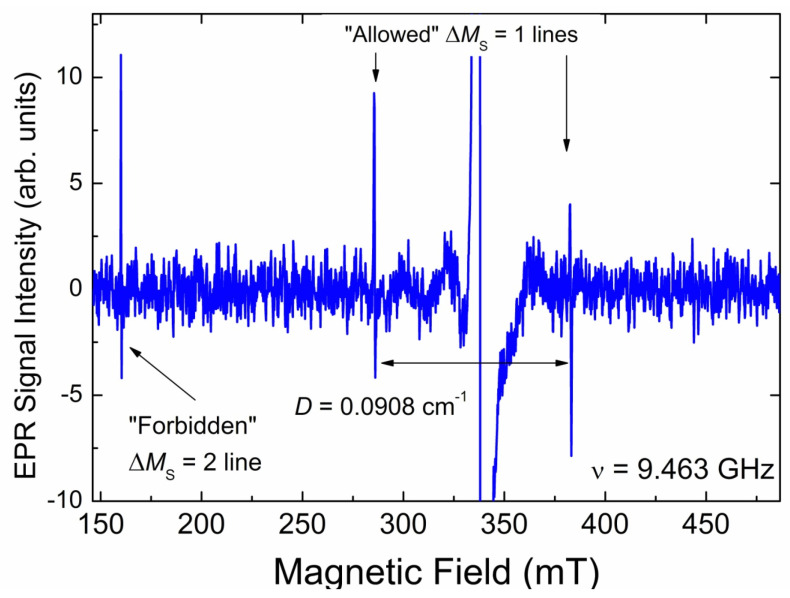
All triplet-related lines (both “forbidden” at half-field and “allowed” on both sides of the intense *g* = 2.00 signals, marked by arrows) in the SP EPR spectrum of the CVD diamond sample. Spectrum was recorded at SR = 19 mT/s, *P*_MW_ = 2 mW, A_m_ = 0.3 mT, and RG = 2 × 10^6^ central signals, with *g* = 2.00 are omitted for clarity. Background signal has been subtracted. Intense *g* = 2.00 line is cut for better presentation of weaker triplet-related lines.

The only EPR signal observed in the *g* = 4 region for the initial CVD diamond sample is a low-intensity asymmetric narrow (∆*H*_pp_ = 0.15 mT) line located at *g*_eff_ = 4.241 ± 0.005. This line is attributed to “forbidden” ∆*M*_S_ = 2 lines in EPR spectra of triplet (*S* = 1) center designated as BS2. The intensity of the “forbidden” line in the pristine CVD sample is found to be sample-dependent. Thus, the content of the triplet centers responsible for this line varies between pieces from ~9 ppb to ~30 ppb. Saturation onset for these centers is at ~20 mW, and the spin–lattice relaxation time was estimated as $T_{eSL}^{forbidden}$~0.32 μs. The half-field line is accompanied by the same weak narrow lines located on both sides of the primary signals’ region (Figure 8). These lines in the polycrystalline EPR pattern of triplets correspond to the “allowed” *x*-, *y*- transitions between Zeeman sublevels. Distance between these low- and high-field lines allows direct determination of zero field splitting *D*, which is found to be *D* = 0.0908 ± 0.0001 cm^−1^. EasySpin simulation of the polycrystalline pattern using *g*_iso_ = 2.0024 and *D* = 0.0908 cm^−1^ provides for the “forbidden” line *g*_eff_ = 4.240, which is in good agreement with the experimentally found value. Attempts to register both the half-field and satellite lines in the FP EPR mode were unsuccessful.

Here, it is worth mentioning that in situ illumination of the CVD samples using white light causes no changes in intensities of “forbidden” and “allowed” lines associated with the BS2 triplet center.

### 3.4. NMR Study of CVD Diamond

**Figure 9:**
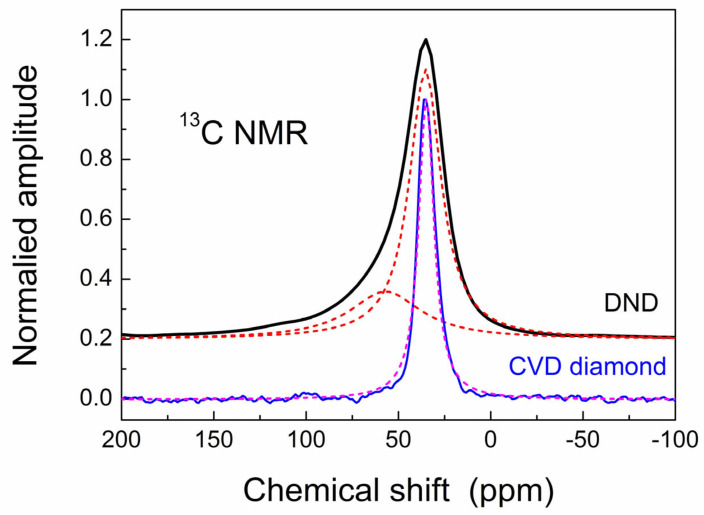
Comparative ^13^C spectra of powdered DND sample and CVD diamond disc. The DND spectrum is deconvoluted into two components. Red and wine dashed lines show Lorentzian fits.

^13^C NMR spectra of bulky CVD diamond disc pieces and, for comparison, of the powder of 5 nm DNDs are shown in Figure 9. One can see that these spectra are quite different. The DND particle consists of a diamond core and a partially disordered shell with a hydrogen-terminated surface [44,45,46,47]. This is reflected in the ^13^C spectrum of DNDs, which is deconvoluted into two components (Figure 9). Our CVD diamond reveals a single ^13^C line, which is much narrower than that in DNDs, meaning a more ordered structure compared to DNDs, and no visible signal of a disordered shell.

**Figure 10:**
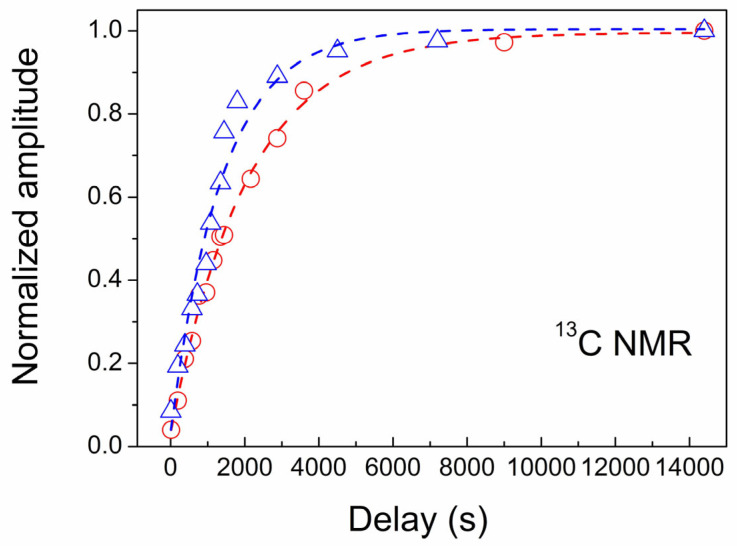
^13^C magnetization recovery of two different samples taken from the same CVD diamond disc shown by red circles (*N*_S_~3 ppm) and blue triangles (*N*_S_~5 ppm). Dashed lines show theoretical fits.

Let us now discuss nuclear spin–lattice relaxation characterized by a time *T*_1n_, at which the energy of the spin system is exchanged with the environment. In the absence of motions with a relatively narrow frequency distribution, such as molecular reorientations, nuclear spin–lattice relaxation in a diamagnetic system occurs due to lattice vibrations that cause energy exchange between the nuclear spin system and lattice phonons. Because the nuclear transition frequencies are small compared to the lattice vibration frequencies, the phonon density at the resonance frequencies is too small to be effective in the nuclear spin–lattice relaxation. The Raman process, in which the difference between the incoming and outgoing phonon frequencies is on the order of NMR frequency, can involve more phonons but is also not effective enough. Therefore, the ^13^C spin–lattice relaxation process in gem diamonds may last from several hours to days. However, the spin–lattice relaxation noticeably accelerates in the case of nuclear relaxation via unpaired electron spins of localized paramagnetic defects due to the strong electron–nuclear interaction and effective heat contact of the electron spins with the lattice modes, which produce an effective channel for the nuclear spin–lattice relaxation. Herewith, according to the theory [48,49], nuclear spin–lattice relaxation time *T*_1n_ is inversely proportional to the density of paramagnetic defects *N*_S_. This is the case with our ^13^C NMR measurements, which show a decrease in relaxation time with an increased number of paramagnetic defects detected by EPR (see Table 2 and Figure 10). While some pieces of our initial CVD diamond with *N*_S_~3 ppm show *T*_1n_ = 2070 s, the relaxation time in the pieces taken from the other location of the CVD diamond disc with *N*_S_~5 ppm of defects is 1.54 times shorter, *T*_1n_ = 1340 s (Table 2 and Figure 10).

We note that nanodiamonds with *N*_S_ = 6.3 × 10^19^ spin/g, or 1256 ppm, show ^13^C spin–lattice relaxation time *T*_1n_ around half a second [44,45,46,47,50,51,52,53].

**Figure 11:**
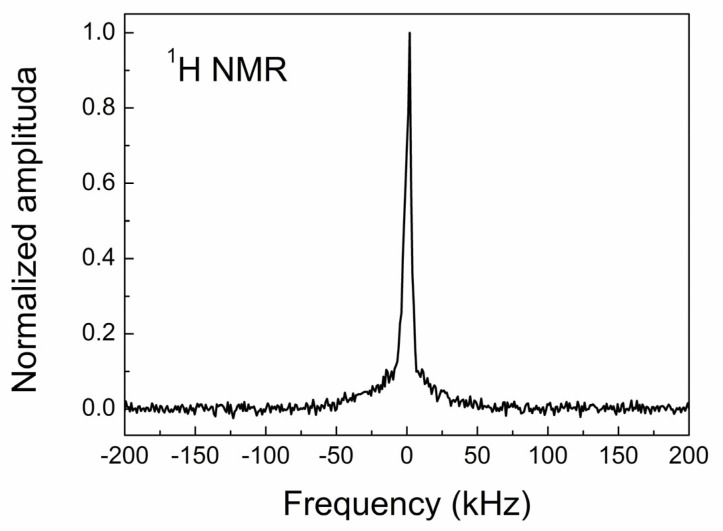
Proton magnetic resonance spectrum of CVD diamond.

When a CVD diamond is deposited from gas-phase mixtures containing hydrogen and methane, its surface is somewhat terminated by hydrogen, forming C–H bonds. In the CVD films, such bonds were previously detected by infrared and NMR spectroscopies [54,55]. Therefore, in addition to the ^13^C NMR measurements discussed above, we also measured the proton magnetic resonance spectrum to check whether such an effect appears in our sample. The result is that our CVD diamond reveals a very weak hydrogen signal (Figure 11) attributed to the surface C–H bonds. This assumes that the hydrogen atoms are not incorporated into the diamond core but are located in grain boundaries and interact with the carbon dangling bonds. This finding is in agreement with the EPR data on the H1 centers.

## 4. Discussion

**Figure 12:**
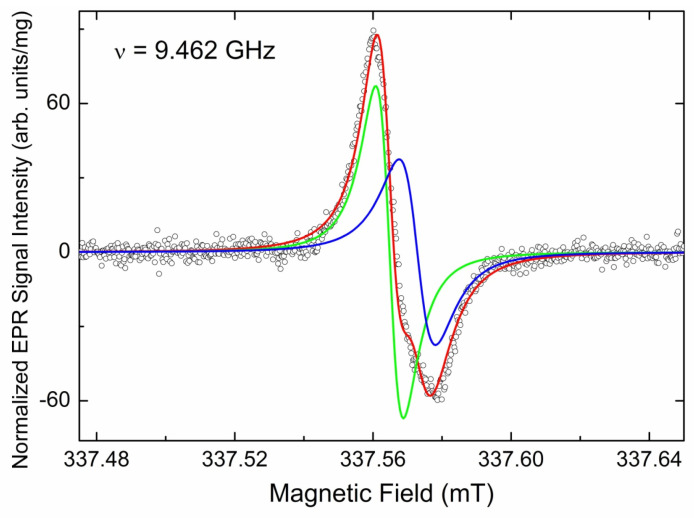
High-resolution RT SP EPR spectrum (open black circles) of the central (*m*_I_ = 0) line in the pristine CVD diamond samples. Spectrum recorded at *P*_MW_ = 200 nW, SR = 0.0477 mT/s, A_m_ = 2 μT, receiver gain RG = 1 × 10^6^, and number of coherent acquisitions n_acq_ = 100, ν = 9.462 GHz. Red trace shows best least square fit of the experimental spectrum using two Lorentzian components, including narrower ∆*H*_pp_ = 0.008 mT (green trace) and broader ∆*H*_pp_ = 0.011 mT (blue trace).

FTIR data indicate the presence of a certain amount (~90 ppm) of nitrogen defects, among them nonparamagnetic aggregated nitrogen pairs (A-centers) and paramagnetic substitutional nitrogen (C-centers, or P1). The combination of CW EPR spectra recorded in both SP and FP modes and progressive power saturation experiments reveals that this bulk CVD diamond sample is rich in various paramagnetic defects. The main paramagnetic species in this sample is substitutional nitrogen N_s_ (P1 centers), which is found to be strongly inhomogeneously distributed over the CVD diamond volume. Moreover, the observed inhomogeneity exists at both macroscopic and microscopic levels. The former is indicated by variations of the P1 (and other primary defects) content between the samples taken from different parts of the bulk CVD diamond disk. The latter becomes clear from the analysis of the saturation behavior of P1-related spectra. Thus, there are at least three kinds of P1 centers distinguished by line width and relaxation behavior, including (i) well-isolated P1 centers responsible for the narrow slow relaxing components in polycrystalline EPR patterns; (ii) clusterized P1 centers responsible for broader faster relaxing components, better observed in FP spectra recorded at higher *P*_MW_ levels (see red and green traces in Figure 4), and (iii) exchange-coupled P1 pairs responsible for the additional hyperfine components with half splitting *A*~1.5 mT (marked by asterisks on the blue trace in Figure 5) [56,57]. A forcible argument in favor of the hypothesis on the inhomogeneous distribution of P1 centers may be drawn from the careful analysis of the line width and shape of the central (*m*_I_ = 0) line of the P1 triplet pattern. The experimental line width for this line was found to be ∆*H*_pp_ = 0.018 mT. Supposing the origin of this line is determined by dipole–dipole interaction between P1 centers and, following the van Wyk model [58], one can predict a line width of ~0.012 mT for the P1 content range 0.6–1.1 ppm found in the sample under study. The line width found (0.018 mT) corresponds to the P1 content above 13 ppm. The latter is higher than the total content of all primary centers in this sample, which does not exceed 5 ppm. Black open circles in Figure 12 represent the high-resolution SP EPR spectrum of the central line. It is clearly seen that the line is slightly asymmetric and most probably consists of two lines differing by their *g*-factors and line widths. The experimental spectrum may be successfully simulated by two overlapping Lorentzian-shaped components (red trace) shifted by ∆*g*~0.00005 and having ∆*H*_pp_ = 0.008 mT (green trace) and 0.011 mT (blue trace), correspondingly. These components may be attributed to slow-relaxing P1 species distinguished by the density of P1 spins in their neighborhood.

In addition to P1 centers, some other nitrogen-related paramagnetic centers were found. Among these, P2 [39,40] and N3V [59] centers may be identified as relatively slow relaxing; in addition, BS1 centers have even shorter relaxation times and are thus unobservable in the FP EPR mode. A small (0.08 mT) nitrogen hyperfine splitting constant found for the BS1 centers points out the interaction of the uncoupled electronic spin (most probably due to vacancy) interacting with the nuclei of two distant nitrogen atoms. None of the previously reported dinitrogen paramagnetic defects (like N1, N4, and W7) satisfy the observation of such a small ^14^N hyperfine constant [39,40]. It allows for the speculation that such a defect has not been previously reported. SP ERP spectra recorded at higher power levels reveal also traces of a slow saturating doublet split by ∆*H*~1.3 mT, (see red trace in Figure 3 and arrows in the zoom panel). These hardly saturating satellite lines may be reliably attributed to “forbidden” hyperfine transitions (∆*m*_I_ = ± 1) in the EPR spectra of H1 centers, which are carbon dangling bonds interacting with hydrogen atoms located in grain boundaries [7].

In an attempt to identify the triplet center BS2, its Spin–Hamiltonian parameters (*g*, *D*) were compared to the same parameters known for a variety of triplet defects previously observed in diamonds [40]. The best match has been found for the W27 triplet center with *g*_iso_ = 2.0025 and *D* = +1794 MHz, which corresponds to *D* = 0.0898 cm^−1^. W27 had been observed in green natural type Ia diamonds and was associated with some nitrogen clusters [60]. In fact, the difference in *D*-values for BS2 (0.0908 cm^−1^) and W27 (0.0898 cm^−1^) exceeds the experimental error in *D*-determination (0.0001 cm^−1^). The same concerns the *g*_eff_-values for the “forbidden” line, which is *g*_eff_ = 4.241 for BS2 and *g*_eff_ = 4.239 for W27 (by EasySpin simulation). These differences allow for the speculation that BS2 is not exactly the same center as W27. On the other hand, the association of BS2 with some nitrogen clusters does make sense, especially taking into account the strongly inhomogeneous distribution of nitrogen-related defects claimed above.

It is important to note that our EPR study revealed no traces of NV^−^ (W15) centers in the CVD diamond sample under study. Moreover, low-fluence e-beam irradiation/annealing and high-fluence gamma irradiation did not create new centers in that nitrogen-containing diamond structure. This strange fact requires further e-beam irradiation/annealing treatment followed by a thorough fluorescent and optically detected magnetic resonance (ODMR) study.

The abovementioned inhomogeneous distribution of the paramagnetic centers over the CVD diamond volume is well-supported by NMR measurements, showing different nuclear spin–lattice relaxation times in two samples taken from different parts of the bulky CVD diamond disc. Herewith the values of the measured relaxation times correlate with the number of paramagnetic centers in these parts measured by EPR.

Let us now discuss the nuclear spin–lattice relaxation in more detail. As shown by Abragam [48] and Goldman [49], nuclear spin–lattice relaxation in solids is mainly caused by the dipole–dipole interaction of nuclear spins with electron spins of paramagnetic defects. Taking into account the dipolar coupling is a short-range interaction and that the concentration of paramagnetic defects in the studied CVD diamond is small (only several ppm), one should explain the nuclear spin–lattice relaxation between the electron spins and distant nuclei. This was performed by Bloembergen [61], who proposed that spin magnetization in a rigid lattice is spatially transferred by nuclear spin diffusion. This mechanism occurs through the mutual flips of neighboring nuclear spins due to interaction terms of $I_{i}^{+}I_{j}^{-}$ type and results in the magnetization transfer from the distant nuclear spins to the localized electron spins. Reynhardt and Hoch [62,63] showed that spin diffusion dominates ^13^C spin–lattice relaxation in diamonds at *N*_S_ < 10 ppm, which is just the case in the question of our samples. Taking into account that the ^13^C spins with natural abundance *n*_c_ = 1.07% are distributed in a random way in the lattice, and using Poisson distribution by putting the probability, they calculated a typical distance $r_{C-C}$ between two carbon spins as *r* = 0.55*n*_c_^−1/3^ = 4.42 Å and the ^13^C nuclei spin diffusion coefficient as $D\approx 7.6\times 10^{-14}cm^{2}s^{-1}$ [63]. The distance over which the spin diffusion can operate is $L=\sqrt{2DT_{1}}$, yielding *L* = 1774 Å for *T*_1n_ = 2070 s. Being applied to the CVD diamond under study, the same approach yields a distance between the paramagnetic defects as 57 and 67.6 Å for *N*_S_ = 5 and 3 ppm, respectively. Thus, spin diffusion can transfer magnetization throughout the ^13^C spin system and induce nuclear spin–lattice relaxation via paramagnetic defects.

## 5. Conclusions

We studied the spectroscopic and structural properties of a thick, sizable CVD diamond disc using EPR, NMR, FTIR, and XRD techniques. The measurements of the as-prepared disc and that after electron and gamma irradiation have been carried out. Detailed investigations of paramagnetic defects have been conducted using a variety of CW EPR techniques. EPR and NMR measurements vote for significantly inhomogeneous distribution of defects across the disk at both macroscopic and microscopic levels. NMR data correlate with the EPR data and reveal nuclear spin diffusion throughout the sample.

## Figures and Tables

**Table 1:** The preparation conditions of the CVD diamond disc.

Stage	Time, h	Pressure, torr	T, °C	Power, W	H_2_, sccm	CH_4_, sccm	O_2_, sccm
Nucleation	60	115	836	4974	500	18	0.5
Growth	56.55	115	836	4974	478	20	2.0

**Table 2:** The density of paramagnetic defects *N*_S_ and spin–lattice relaxation times *T*_1n_ in two different parts of the CVD diamond disc CVD-1 and CVD-2.

Sample	*N*_S_, ppm	*T*_1_, s
CVD-1	3	2070 ± 79
CVD-2	5	1340 ± 126

## Data Availability

Data are contained within the article.
